# A Novel *Rhipicephalus microplus* Estrogen Related Receptor (RmERR), a Molecular and In Silico Characterization of a Potential Protein Binding Estrogen

**DOI:** 10.3390/microorganisms11092294

**Published:** 2023-09-12

**Authors:** Hugo Aguilar-Díaz, Rosa Estela Quiroz-Castañeda, Ixchel Guadalupe Díaz-Esquivel, Raquel Cossío-Bayúgar, Estefan Miranda-Miranda

**Affiliations:** Centro Nacional de Investigación Disciplinaria en Salud Animal e Inocuidad, INIFAP, Carretera Federal Cuernavaca-Cuautla No. 8534, Jiutepec 62550, Morelos, Mexico; requiroz79@yahoo.com.mx (R.E.Q.-C.); ixcheldg341@gmail.com (I.G.D.-E.); cossio.raquel@inifap.gob.mx (R.C.-B.); miranda.estefhan@inifap.gob.mx (E.M.-M.)

**Keywords:** hormone receptors, transregulation, endocrine, phylogenetics, tick, estrogen, *Rhipicephalus microplus*

## Abstract

The search for targets to control ticks and tick-borne diseases has been an ongoing problem, and so far, we still need efficient, non-chemical alternatives for this purpose. This search must consider new alternatives. For example genomics analysis is a widely applied tool in veterinary health studies to control pathogens. On the other hand, we propose that regulation of endocrine mechanisms represents a feasible alternative to biologically controlling tick infestations. Thus, we performed the molecular identification of an estrogen-related receptor gene of *Rhipicephalus microplus* called RmERR by RT-PCR in tick ovaries, embryonic cells, and hemolymph, which allowed us to analyze its expression and propose potential functions in endocrine mechanisms and developmental stages. In addition, we performed an in silico characterization to explore the molecular interactions of RmERR with different estrogens, estrogenic antagonists, and endocrine disruptor Bisphenol A (BPA), finding potential interactions predicted by docking analysis and supported by negative values of ΔG (which suggests the potential interaction of RmERR with the molecules evaluated). Additionally, phylogenetic reconstruction revealed that RmERR is grouped with other tick species but is phylogenetically distant from host vertebrates’ ERRs. In summary, this study allowed for the identification of an ERR in cattle tick *R. microplus* for the first time and suggested its interaction with different estrogens, supporting the idea of a probable transregulation process in ticks. The elucidation of this interaction and its mechanisms unveiled its potential as a target to develop tick control strategies.

## 1. Introduction

Ticks as blood feeders and vectors of pathogens to humans and animals, including livestock, should be controlled to avoid damages caused by infestations [1,2]. Currently, the alternatives used to control ticks’ infestations are based on chemical compounds acting as acaricides. However, their use generates severe environmental pollution and induces resistance in tick populations [3,4]. Therefore, the success of novel acaricides depends on making them contaminant-free, with a high potential to inhibit essential tick organs and functions, including the nervous system (synganglion), which is considered adequate since it controls all of the tick systemic functions [5]. Along with the nervous system, the immune and endocrine systems are present in all vertebrates, acting as a coordinated macro-system called neuro-immuno-endocrine; and recently, its presence has been considered essential to cell signaling in vital processes of invertebrates; however, the mechanisms of action are unknown [6]. So far, studies about insects and crustaceans have been the base for what we know about the molecular endocrinology of the Arthropoda [7,8]. Currently, tick endocrinology research assumes that ticks regulate their larval, nymphal, and adult development by the same hormones and mechanisms that insects use; however, this does not necessarily occur [7]. Interestingly, the juvenile hormone (JH), which participates in insect molting and development, has not been identified in ticks [9].

In contrast, some hormones and their cellular receptors have been identified in ticks, including molting and neuro hormones (ecdysteroids), involved in molting, development, and reproduction (vitellogenesis) [10,11,12,13,14]. For example, during the life cycle and after engorgement, ecdysteroid hormones trigger the salivary glands degeneration in *Rhipicephalus haemaphysaloides* [12]. There even exist reports proposing that the tick microbiome is responsive to the hormone 20-hydroxyecdysone, which regulates vitellogenesis and the egg development in *Dermacentor variabilis* [9]. Finally, the recent advances in tick genomics, transcriptomics, and proteomics have allowed researches to explore other possibilities to understand the endocrine and molecular host-vector-pathogen relationships [15,16,17,18,19]. In this regard, transcriptomes from the ovary, salivary glands, fat body, and midgut of *Rhipicephalus microplus* revealed that eclosion hormone and allatostatins are overexpressed during pupal-adult ecdysis, and the *Ixodes scapularis* synganglion transcriptome, showed the presence of many neuropeptides and hormones receptors, and steroidogenic acute regulatory proteins [20].

On the other hand, parasitism is a symbiosis in which the parasite relies entirely on hosts for access to resources, as in the case of ticks that may use host blood components for their benefit [21,22]. An example is the use of hemoglobin as an exogenous source of heme group that the tick requires as part of its diet and for reproduction [23], or when the same protein is fragmented to form small molecules with antimicrobial activity or complement system proteins to regulate the tick gut microbiota populations [24]. In addition, a mechanism of host exploitation called trans-regulation has been described in parasites. This process demands that the parasite develop similar structures (receptors) to those of the host to benefit directly from the host’s hormones or growth factors, enabling rapid establishment, increased growth, and reproduction rates [25,26]. Despite the limited evidence of steroid receptors in invertebrates, some binding proteins for vertebrate steroids have been reported in nematodes, echinoderms, mollusks, and crustaceans [27].

In this regard, in recent years it has been proposed that some vertebrate hormones trigger signaling processes in invertebrates, known as heterologous hormonal actions [28]. Following this idea, the characterization of several hormone receptors in invertebrates has been reported, such as estrogen receptors (ERs) and estrogen-related receptors (ERRs) [29,30]. Studying these receptors is an approach to understanding their functional role and relation with their vertebrate orthologues [31]. Although ERs are significant regulators of development, behavior, and reproduction in vertebrates, much less is known about how they regulate physiology and behavior in invertebrates [32]. So far, it is known that ERRs belong to the orphan nuclear receptor (ONR), a subgroup of the nuclear receptor family whose ligand has not yet been identified [33].

Regarding ERRs, they have a role in physiologic and reproductive processes in vertebrates, such as controlling and fine-tuning the expression of steroidogenic genes that regulate testicular steroidogenesis, as has been observed in mammalian cells cultures [34]. They also participate in placental development and control of lipid metabolism [35], and is proposed that modulate the levels of estrogens and the expression of estrogen-regulated genes in their target tissues [36,37]. At the same time, some research in invertebrates suggests that ERR could be involved in the reproduction of some Arthropoda, such as *Drosophila melanogaster* ERR which regulates metabolic processes supporting larval growth and cell proliferation [38,39,40]; in Crustacean *Crassostrea gigas*, ERR is dynamically expressed during developmental with emphasis at pre-metamorphic and metamorphic stages [41,42]; while in Mollusk, some ERR have been reported but with an unknown function so far [31].

## 2. Materials and Methods

### 2.1. Identification of the R. microplus ERR (RmERR) Gene Sequence

Based on the possible occurrence of a transregulation process, we hypothesized that the ER from tick and its host *Bos taurus* could probably share some sequence similarity. So, the following strategy was using *B. taurus* estrogen receptor sequence 1 (ESR1, NCBI, Protein ID NP_001001443.1) as a query and performed a BLASTp in ticks (taxid:6935) in NCBI database (National Center for Biotechnology Information, Bethesda, MD, USA) [43]. The matched sequence retrieved was an *R. microplus* ERR (NCBI, Protein ID XP_037272566.1), from which we obtained its gene sequence (NCBI, Gene ID 119164474) in “Download datasets”.

### 2.2. Strains and Cell Lines of R. microplus

The tick strain Media Joya-INIFAP was used to carry out infestations on a bovine, applying 10,000 15-day-old larvae kept in the laboratory at 28 °C with constant humidity. At 21 days post-infestation, engorged or semi-engorged females were collected and washed 3X with distilled water to remove hair and skin from the host. Three washes are carried out by immersion in 10% benzalkonium chloride for 10 min at room temperature and rinsing 3X with sterile distilled water. A final wash was performed with sterile distilled water and an antibiotic-antimycotic mixture (100X, Thermo Fisher Scientific, Waltham, MA, USA) at a ratio of 1:100 for 10 min at room temperature and two rinses with sterile distilled water. Ticks were dried on a bed of sterile gauze. The embryonic cells from *R. microplus* strain Su-INIFAP [44], were cultured on a 25 cm^2^ cell culture flask (Corning, Corning, NY, USA) in 5 mL of Leibovitz’s L-15 -MEM (1:1) (Thermo Fisher Scientific) supplemented with 20% fetal bovine serum (Gibco) and antibiotic-antimycotic mix (100X, Thermo Fisher Scientific) and incubated at 32 °C and 3% CO_2_. The cells in the log growth phase were harvested and washed by centrifugation at 8000× *g* in PBS (Sigma, St. Louis, MO, USA) and quantified (Nabi-UV/Vis Nano Spectrophotometer, MicroDigital, Seoul, Republic of Korea) before RNA extraction.

### 2.3. Tissue and Hemolymph Extraction

The ovaries were extracted by excision (10 female ticks) with a sterile scalpel and precision flat-tip tweezers, according to Cossío-Bayúgar et al. [45]. The tissue was pooled and stored at −20 °C before RNA extraction. The hemolymph was extracted from 15 female ticks by dorsal hipocuticular puncture according to the protocol reported by Aguilar-Díaz et al. [46]. 500 µL of hemolymph (hemocytes) were quantified in a cytosmart cell counter (Corning^®^, Corning, NY, USA) before RNA extraction.

### 2.4. RNA Extraction and cDNA Synthesis

30 mg of ovary tissue were weighted and ground in liquid nitrogen to a fine powder with a mortar and transferred to a sterile microcentrifuge tube. On the other hand, a total of 5 × 10^6^ embryonic cells and hemolymph (hemocytes) were incubated separately in the FARB buffer. The total RNA of all samples was purified with a FavorPrep Tissue RNA purification kit (FavorGen Biotech, Wien, Austria). The total RNA obtained was quantified in Nabi-UV/Vis Nano Spectrophotometer, and the Reverse Transcriptase (RT)-PCR was immediately carried out. The cDNA was synthesized following the kit GoScriptTM^®^ Reverse Transcription System (Promega, Madison, WI, USA) instructions using one µg of total RNA. The cDNA was stored at −20 °C until use.

### 2.5. Primer Sequences

To assess the expression of *RmERR* by PCR, a pair of primers was designed based on the sequence of *R. microplus* retrieved in KEGG BLASTN (Kyoto Encyclopedia of Genes and Genomes, Kyoto, Japan). The primer sequences were: RmERR-F: 5′-AGAAGTGCCTCAAGATGGG-3′ and RmERR-R: 5′-TCACCGCGTGAGCGTGTCG-3′. PCR amplified the constitutive gene phospholipid-hydroperoxide glutathione peroxidase (*PHGPx*) with the sequences of the primers: PHGPx-F: 5′-GCTAGACTGCACAAGCAATACGGG-3′ and PHGPx-R: 5′-GTTTGCAGACACCTCAGCGTGCC-3′.

### 2.6. Relative Expression of RmERR and PHGPx

A PCR reaction was performed using 500 ng of cDNA of ovaries (Ov), embryonic cells (Ec), and hemolymph (Hl) as templates. The reaction of 25 µL contained 12.5 µL of 2X GoTaq^®^ Green Master Mix (Promega), 0.25 µL of each primer 10 µmol/µL. PCR amplification was performed under the following conditions for both genes: initial denaturation at 94 °C, 5 min; 30 cycles of denaturation at 94 °C, 1 min; annealing at 61 °C, 30 s; and extension at 72 °C, 30 s; and a final extension at 72 °C 10 min. A 2% agarose gel was stained with 20 µL of ethidium bromide, and electrophoresis was performed at 120 V for 25 min. The PCR products were excised from the gel, purified using kit Wizard^®^ SV Gel and PCR Clean-Up System (Promega), and sequenced at the SENASICA sequencing unit. A one-way ANOVA was performed in GraphPad Prism 9 for Windows GraphPad 7 Software, Boston, MA, USA, www.graphpad.com, accessed on 11 July 2023. The densitometric analyses were carried out with the information of the calculated area and pixel value statistics of user-defined selections in ImageJ 1.8.0 software, accessed on 5 July 2023 [47]. 

### 2.7. Bioinformatics Analysis

The prediction of available domains and structural features of RmERR was assessed in the Conserved Domains Database (CDD, NCBI) [48]. The multiple alignments of sequences of human estrogen receptors (ER) and estrogen-related receptors (ERR) and RmERR were performed in Clustal Omega (Cambridgeshire, UK) [49]. The sequences used were: ERα (SwissProt, P03372.2); ERβ (SwissProt, Q92731.2); ERRα (GenBank, KAI4072039.1); ERRβ (GenBankKAI4061772); and ERRγ (GenBank, KAI4084975). The alignment was visualized in Jalview 2.11.2.7 (Scotland, UK) [50].

### 2.8. Phylogenetic Analysis of RmERR

A phylogenetic tree was reconstructed with the protein sequences of estrogen-related receptors that included ERRα, ERRβ, and ERRγ, reported in the GenBank database. Invertebrates including the Classes Insecta: XP_02171296.2, XP_02971378.1, XP_039452504.1, BAE96770.1, XP_040228627, NP_729340, NP_001155988.1, XP_011057174.1, ACW84414.1, XP_019880246.1, XP_023711770.1, XP_021941221.1, XP_013170855.1, XP_021196875.1; Crustacea: KAB7506312.1, AXU37748.1, ADB43256.1, MPC32341.1, KAG7160291.1, XP_037789095, XP_042872270.1. XP_037789096.1, XP_042872273.1; and Arachnida: XP_046919025.1, OTF76932.1, RWS30537.1, GBM86989.1, XP_015925325.1, XP_029847411.3, XP_029847410.3, XP_037581544.1, L7LXI7, XP_037525182.1, 119164474K08703, KAH8031228. For Vertebrates including species: AAH63795.2, XP_016872802.1, AAS20260.1, XP_011285297.1, NP_001178302.1, ALS35334.1, AAB51250.1, XP_027688314.1, XP_009275111.2, XP_0081177296.1, XP_028580467.1, NP_001127757.1. ALS35337.1, XP_013872664.1, XP_024256361.1, AAC99409.1, ALS35336.1, AAI32598.2, XP_028574920.1, XP_009279003.1, XP_015143195.1, XP_027493631.1. A phylogeny of ERRs was reconstructed with the Neighbor-joining method and 1500 bootstrap replications implemented in software MEGA 11.0.11 (Philadelphia, PA, USA) [51]. The multiple alignments were performed with Clustal Omega and visualized in Jalview.

### 2.9. Three-Dimensional (3D) Modeling of RmERR

To obtain the RmERR 3D structure model, we used AlphaFold (AlphaFold2_advanced) (Cambridgeshire, UK) an AI system developed by DeepMind that predicts a protein’s 3D structure from its amino acid sequence [52]. The number of predicted models recommended by AlphaFold was five, the max recycles set to 48, the tolerance was set to five, and the number of samples was set to one. The confidence measure of the model is called pLDDT and corresponds to the model’s predicted score on the lDDT-Cα metric, which produces a per-residue estimate of its confidence on a scale from 0–100. The 3D structures were visualized with UCSF Chimera version X software (San Francisco, CA, USA) [53].

### 2.10. RmERR Molecular Docking

Molecular docking was conducted to evaluate the possible interactions between the RmERR 3D structure, and different ligands in CB-DOCK2 [54]. The structures of (1) estrogens: 17β-estradiol (5757); estrone (698), and estriol (5756); (2) synthetic estrogen: diethylstilbestrol (DES, 448537); (3) endocrine disruptor; bisphenol A (6623); and, (4) estrogenic antagonists: tamoxifen (2733526), and hydroxytamoxifen (449459) were obtained from the PubChem database (https://pubchem.ncbi.nlm.nih.gov/) accessed on 1 June 2023. The top-ranked docking parameters were predicted using affinity and Vina-score values. The conformations with the lowest docking scores (highest affinity) were selected and visualized via Chimera version X software.

## 3. Results

### 3.1. Identification of the R. microplus ERR (RmERR) Gene Sequence

Once the BLASTp was performed, the best hit obtained using *B. taurus* ER sequence (ESR1, NCBI, Protein ID NP_001001443.1) as query retrieved an *R. microplus* ERR (NCBI, Protein ID XP_037272566.1), from which we obtained the gene sequence (NCBI, Gene ID 119164474) of 1467 nt (489 aa) in “Download datasets”.

### 3.2. Relative Expression of RmERR

**Figure 1:**
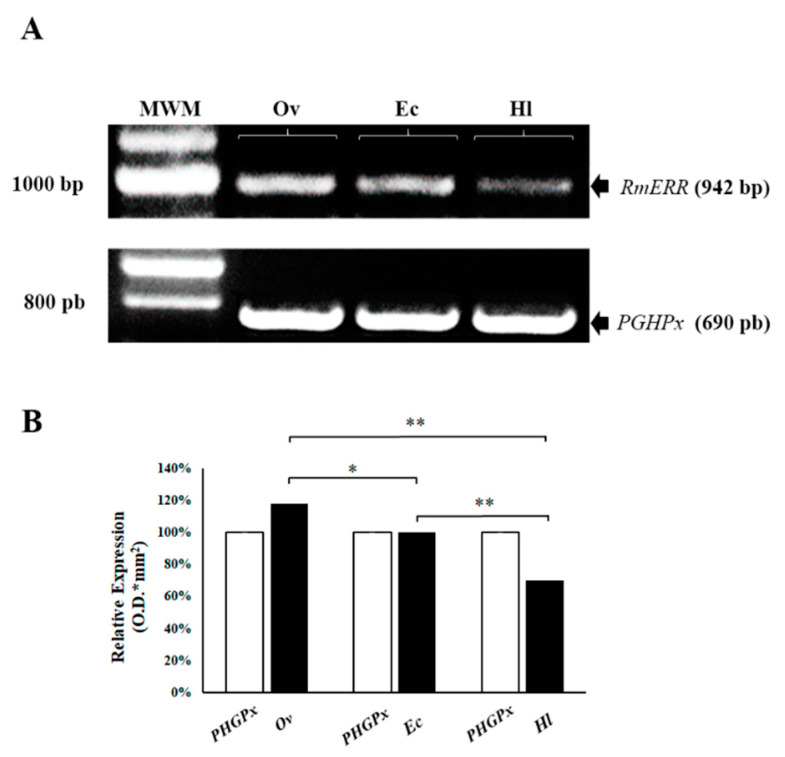
**Relative expression of *RmERR* gene in ovaries (Ov), embryonic cells (Ec), and hemolymph (Hl).** (**A**) A differential expression by RT-PCR of *RmERR* (942 bp) Ov, Ec, and Hl was observed; the constitutive gen was *PGHPx* (690 bp). (**B**) A densitometry analysis showed an overexpression of 18% in Ov; no change in expression in Ec; and an under-expression of 30% in Hl. Significant differences are shown with an asterisk (*p* ≤ 0.05) or two asterisks (*p* ≤ 0.01) by ANOVA test. MWM: Molecular Weight Marker.

We performed a reverse transcriptase-PCR assay (RT-PCR) to determine the expression of the *RmERR* gene. The results showed a relative differential expression between the Ov, Ec, and Hl samples (Figure 1A). Likewise, densitometry analyses revealed an 18% overexpression of *RmERR* in ovaries, no change in expression in embryonic cells, and a 30% underexpression in hemolymph compared to the expression of the constitutive *PHGPx* gene. Additionally, the ANOVA statistical analyses revealed significant differences between the experimental groups (Ov-Ec, *p* ≤ 0.05; Ov-Hl, *p* ≤ 0.01; and Ec-Hl, *p* ≤ 0.01) (Figure 1B).

### 3.3. Bioinformatics Analysis

**Figure 2:**
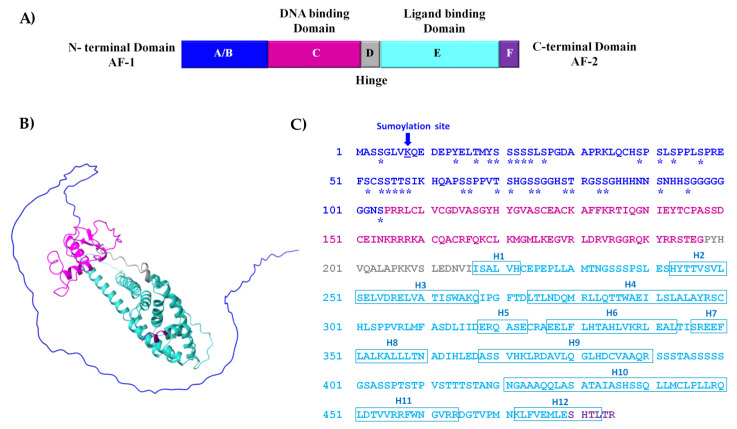
**Structure of estrogen-related receptor of *R. microplus* (RmERR).** (**A**) Diagram structure shows six conserved regions (A/B, C, D, E, and F domains); (**B**) the predicted 3D structure shows the conserved regions; and (**C**) the 485 aa sequence, showing post-translational modifications phosphorylation (asterisk) and sumoylation (arrow) in the N-terminal domain. The position of the 12 helixes (H1 to H12) is presented in blue squares in the ligand binding domain.

The CDD analysis of the 485 amino acids RmERR sequence showed six regions that correspond to an N-terminal domain (A/B), a DNA binding domain (C), the hinge region (D), the ligand-binding domain (E), and a C-terminal domain (F) (Figure 2A). This organization from regions A to F is a typical arrangement in vertebrates’ estrogen-related receptors. The RmERR 3D structure showed the 12 helixes predicted in the ligand-binding domain and an N-terminal AF-1 domain (Figure 2B). Additionally, Figure 2C shows the RmERR primary sequence and the amino acids corresponding to the 12 predicted helixes and sumoylation and phosphorylation sites.

**Figure 3:**
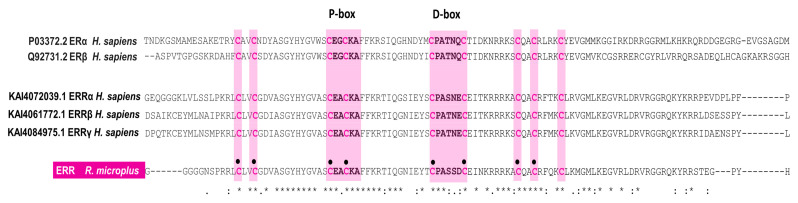
**RmERR DNA-binding domain features.** The RmERR binding domain contains eight (black circles) out of nine cysteines (pink letters) that bind to zinc ions in the zinc finger domain. The P-box shares the same sequence of human ERR, while the D-box contains a variant of the sequence reported for human ER and ERRs. Asterisks indicate positions which have single, fully conserved residue, indicates conservation between groups of strongly similar properties, and, indicates conservation between groups of weakly similar properties.

Interestingly, when we compared human ERR and RmERR DNA binding domains, we identified a different D-box sequence in RmERR (CPASSDC), whereas the P-box sequence (CEACKA) is identical. On the other hand, the comparison of P-box and D-box showed different sequences in human ER and RmERR. However, these variants contained the conserved cysteine residues flanking each box. Additionally, in all sequences (human ER, ERR, and RmERR), we identified two cysteine residues localized in the proximal region of the P-box and three cysteine residues near the D-box. This eight-cysteine arrangement is responsible for interaction with DNA and is considered a potential zinc-binding site (Figure 3).

**Figure 4:**
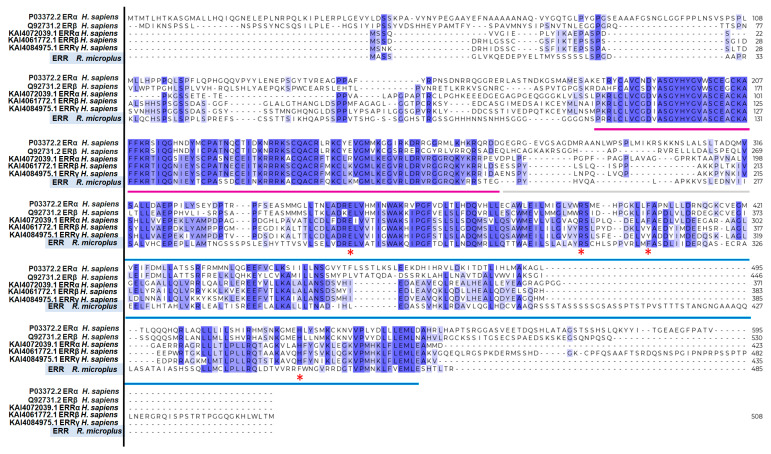
**Multiple alignments of human ER, ERR, and RmERR.** The sequences are conserved in DNA-binding domain (magenta line), the hinge (grey line), and ligand-binding domain (blue line). The identity percentage of whole sequences of RmERR with ER alpha and beta is 33.33 and 32.78%, respectively. In contrast, the identity of RmERR with ERR alpha, beta, and gamma is 50.25, 44.87, and 45.37%, respectively. The identity percentages of the single amino acids is shown from violet (fully conserved residue), mauve (residues of groups of strongly similar properties), and lilac (conserved residues between groups of weakly similar properties). The red asterisks show the four amino acids that binds to estradiol in human ERs and ERRs (E, R, F/Y, and H) and the changes (E, R, F, and F) in RmERR.

We performed a multiple alignment to assess the identity of RmERR with human ER and ERR sequences. The results showed the identity percentage of RmERR with ER alpha and ER beta of 33.33 and 32.78%, respectively. In contrast, the identity percentages of RmERR with ERR alpha, beta, and gamma increased to 50.25, 44.87, and 45.37%, respectively (Table S1). It is important to note that the identity was mainly in the DNA and ligand binding domains, and some variations in the N-terminal and C-terminal domains were observed. Even more significant is the fact that the ligand binding domain in RmERR contains three (Glu257, Arg298, Phe310) out of four amino acids in the pocket that binds estradiol in human ERα and β (Glu353, Arg394, Phe404, and His524); interestingly, the fourth amino acid changed from His524 to Phe458 (Figure 4).

### 3.4. Phylogenetic Analysis of RmERR

**Figure 5:**
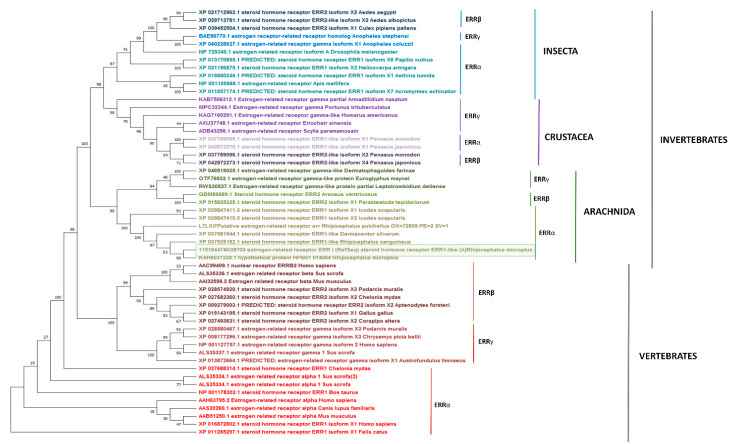
**Phylogenetic reconstruction of RmERR.** The evolutionary history was inferred using the Neighbor-Joining method. The optimal tree is shown. The percentage of replicate trees in which the associated taxa clustered together in the bootstrap test (1000 replicates) are shown next to the branches. The tree is drawn to scale, with branch lengths in the same units as those of the evolutionary distances used to infer the phylogenetic tree. The evolutionary distances were computed using the JTT matrix-based method and are in the units of the number of amino acid substitutions per site. There were a total of 948 positions in the final dataset. Evolutionary analyses were conducted in MEGA11.

The neighbor-joining tree was constructed with representative ERRα, ERRβ, and ERRγ protein sequences of Invertebrates (Classes Insecta, Crustacea, Arachnida), and Vertebrates from diverse taxa of Mammals, Birds, Reptiles, and fishes previously deposited in the Genbank. Our results showed that RmERR is nested within a cluster including other ticks (*I. scapularis*, *R. sanguineus*, *Rhipicephalus pulchellus*, and *D. silvarum*) ERRα with a solid bootstrap support of 100. Additionally, in invertebrates, ERRs of Insecta and Crustacea were more closely related than Arachnida ERRs. As expected, the Invertebrates ERRs are separated from Vertebrates ERRs as suggested by the bootstrap support of 98 (Figure 5).

### 3.5. RmERR Three-Dimensional Modeling

**Figure 6:**
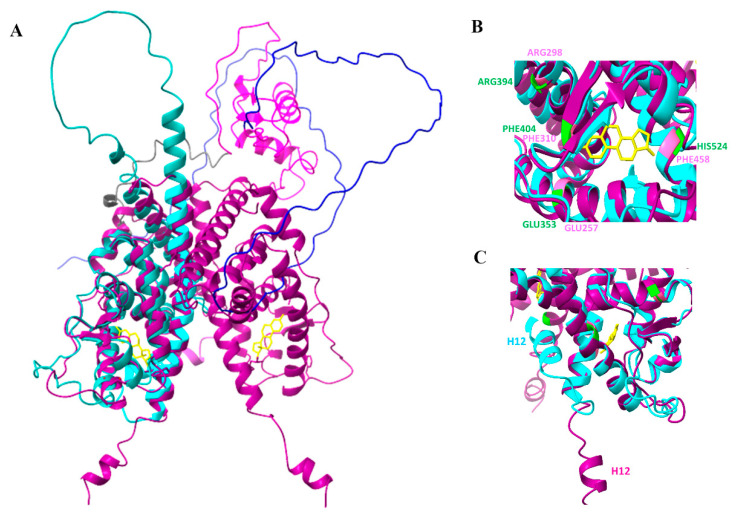
**RmERR 3D modeling.** (**A**) Superimposition of RmERR monomer (cyan) on ERα dimer (magenta, PDB:1A52). In 1A52, the estradiol (yellow) is located in the ligand binding domain; (**B**) Superimposition of the estradiol binding pocket of 1A52 (amino acids in green) and RmERR (amino acids in rose); (**C**) The helix 12 (H12) locates near the estrogen binding site and shows a different spatial arrangement. The RMSD (root mean square deviation) between the two structures is 1.089 Å.

The confidence measure of the five models retrieved had regions with pLDDT > 90 which are modeled with high accuracy. The monomeric predicted model rank 1 of RmERR was superimposed on the dimeric ERα ligand binding domain complexed to estradiol (PDB: 1A52) to compare the 3D structure and identify the potential estradiol binding site. The superimposition revealed a high structural similarity between the RmERR ligand binding domain and the same domain in 1A52, including the estradiol binding pocket (Figure 6A). According to the structure of 1A52 3D, estradiol binds to the residues Glu353, Arg394, Phe404, and His524; interestingly, the amino acids Glu353, Arg394, Phe404 matched with Glu257, Arg298, and Phe310 of RmERR, respectively. However, in RmERR, the His524 changed to Phe458 (Figure 6B). Additionally, the RmERR 3D structure showed that the last 12 amino acids (KLFVEMLESHTL) that correspond to helix 12 (H12) presented a different spatial arrangement in comparison to that of 1A52 (Figure 6C).

### 3.6. Docking of RmERR

**Figure 7:**
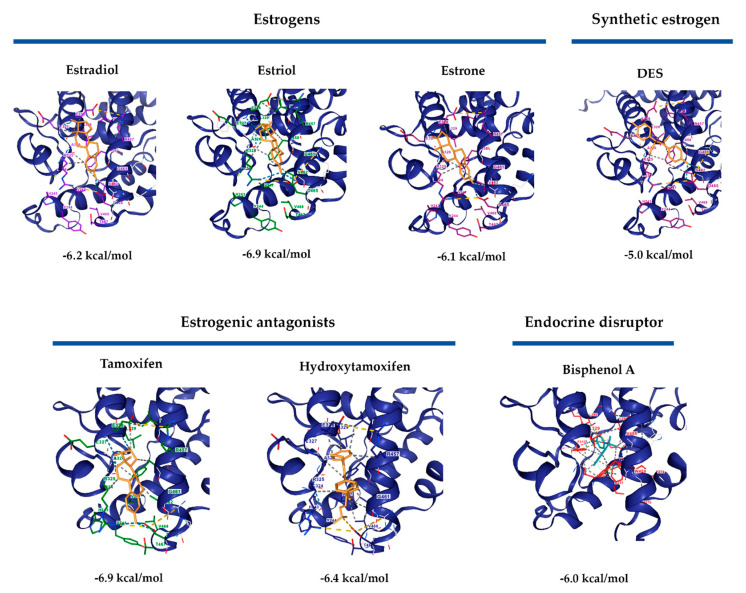
**RmERR Molecular docking with estrogens, synthetic estrogen, estrogenic antagonists, and endocrine disruptor.** All ligands showed an interaction with RmERR according to the ΔG values. Interestingly, the interactions with the higher values (most negatives) of ΔG are estriol and tamoxifen, while estradiol shows a ΔG of −6.2 kcal/mol. Amino acids interacting with ligands are shown in different colors.

A molecular docking analysis was performed to assess the possible RmERR interactions with estrogens (estradiol, estriol, and estrone), synthetic estrogens (DES), estrogenic antagonists (tamoxifen and hydroxytamoxifen), and endocrine disruptors (bisphenol A). Our results predicted molecular interactions of these ligands with RmERR according to Vina score values obtained for each docking (Figure 7). The RmERR docked with estrogens and synthetic estrogen showed the binding amino acids (Arg325, Ala326, Glu327, Glu328, Arg457, Phe458, Gly461, Val462, and Asp465) that potentially would establish different molecular interactions in the RmERR ligand binding domain (Figure 7, upper panel). On the other hand, the docking of RmERR with estrogenic antagonist revealed molecular interactions with tamoxifen mediated by amino acids Arg325, Ala326, Glu327, Glu328, Leu329, Val454, Arg457, Phe458, Gly461, Val462, Arg464, and Asp465. At the same time, the predicted residues that would be interacting with hydroxytamoxifen are His243, Val247, Cys324, Arg325, Ala326, Glu327, Glu328, Leu329, Val454, Arg457, Phe458, Gly461, Val462, Arg464, Asp465, Thr467, and Val468 (Figure 7, lower panel). In addition, the docking results also suggested a molecular interaction of the endocrine disruptor bisphenol A with RmERR via the amino acids Leu250, Leu253, Val254, Glu257, Trp287, Ala288, Leu291, Leu295, Arg298, Met309, Phe310, Ala311, Ala326, Leu329, Val455, and Phe458 (Figure 7, lower panel). It is essential to highlight that Phe458 is the typical amino acid in the molecular interactions of all dockings. 

## 4. Discussion

The steroid hormone estrogen plays modulatory roles in physiological and pathophysiological human processes. Estrogens perform their role by interacting with estrogen receptors (ERs), which are ligand-dependent transcription factors that activate transcriptional processes and/or signaling cascades resulting in the control of gene expression [55,56]. Interestingly, only a few ER and ERR coding genes have been identified or genomically sequenced in different invertebrate species, and their presence has suggested that they could have some correlations with reproduction and sex differentiation [29,34,39]. However, there has been no experimental test so far. In mollusks, such as bivalves, cephalopods, and gastropods, the identified ER and ERR orthologues have shown structural similarity to vertebrates’ ERs [57]. Therefore, the characterization of a possible estrogen receptor with a role in biological processes in tick *R. microplus* was the aim of this work, and here, we present for the first time the molecular and sequence identification of RmERR and propose the possibility of interacting with estrogenic ligands. In this regard, the RT-PCR assay showed differences at the gene expression level of *RmERR*; the ovaries showed higher expression than embryonic cells and hemolymph. This fact supports the idea that RmERR may probably participate by regulating development processes, such as in fish *Fundulus heteroclitus* where the expression of ERR in the ovary is related to its reproduction [58]. Additionally, reports stated that vitellogenesis could be regulated by estrogenic compounds [59]. Nevertheless, in *R. microplus* this finding requires more in-depth studies and analyses.

On the other hand, the expression of *RmERR* in Ec would suggest its participation in cells of early stages of tick development. Although the role of RmERR in these cells has not been determined, some reports in invertebrates have suggested the participation of estrogen receptors in larval development by a mechanism mediated by estrogen [31]. Lastly, the expression of *RmERR* in hemolymph may indicate a potential role in the calcium (Ca^2+^) regulation associated with the immune response. As it is known, the hemocytes are the cellular component of the immune response in ticks. In this regard, Canesi et al. [60] found that estradiol induced a significant increase of cytosolic Ca^2+^ in hemocytes of the mussel *Mytilus galloprovincialis* and proposed that the mechanisms of action of estradiol may play a key role in immune endocrine interactions in invertebrates. Therefore, we hypothesize that if the RmERR can bind E2, we cannot discard a potential immune response in ticks hemocytes mediated by RmERR, estradiol, and the changes in Ca^2+^ concentration.

We found that RmERR has the classical arrangement for hormone receptors that comprises six domains: A/B, C, D, and E/F, from amino to carboxyl terminal end, previously reported for vertebrates ER and ERR. The N-terminal domain (A/B) is also known as the activation domain (AF-1) and has the characteristics of ligand-independent transcriptional activation. The transcriptional activity of this domain could be regulated by post-translational modifications (phosphorylation and sumoylation), where Lys8 is a predicted site for sumoylation and one tyrosine, four threonines, and twenty serines are potential phosphorylation sites, as in other ERRs have been reported [61,62]. In the DNA binding domain (C), we identified the reported residues (CEACKA) of the proximal box (P-box) in the first zinc finger domain. The amino acids Glu203, Gly204/Ala204, and Ala207 of this domain are critical for sequence discrimination and binding to the DNA of the responsive genes. Previous reports indicate that the DNA binding domain is present in ER and ERR to recognize either estrogen-related response elements (ERRE) in promoters of some estrogen-responsive genes (aromatase and lactoferrin) or estrogen-response elements (ERE) in promoters of genes encoding proteins involved in embryonic development (vitellogenin A2, cathepsin D, and progesterone receptors). Also, the distal box (D-box) that participates in the dimerization interface between two ERR monomers was identified in the second zinc finger domain, with the sequence CPASSDC, which is a variant of the sequence reported for human ERRs (CPASNEC/CPATNEC) [63,64,65,66]. The amino acid variation resulted in a conservative substitution, which would not affect the structure of the monomer binding site. In contrast, the conserved arrangement of the eight cysteines that bind to zinc ions in the zinc finger domains was identical in all ER, ERR, and RmERR sequences.

We also identified the hinge region (D) separating the DNA-binding domain from the ligand-binding domain (E); the conservation of the amino acids in the hinge region is low (25% to 30%) when compared with human ER and ERR as it has been reported May et al. [63]. In addition, the ligand-binding domain (E) is the largest in the receptor structure and comprises 12 helixes. An important fact is that the amino acids that bind estradiol are highly conserved between the RmERR and human ERs and ERRs, suggesting a probable interaction with other estrogenic molecules. In this regard, we found that three out of four amino acids binding estradiol are conserved in RmERR. Lastly, the predicted C-terminal domain (F) of RmERR comprises the short sequence SHTLTR. According to May et al. [63], ERRα and ERRγ lack an F domain, and probably RmERR lacks this C-terminal domain since the sequence is too short. Intending to elucidate the 3D structure of RmERR, we performed modeling based on artificial intelligence in AlfaFold. The predicted model of RmERR was superimposed in the Erα structure (PDB: 1A52) to identify a possible estradiol binding site. When we compared the conserved amino acids that bind estradiol (Glu353, Arg394, Phe404, His524) in the human E domain (1A52) with the RmERR E domain, interestingly, we found that three amino acids, Glu257, Arg298, Phe310, are conserved and superimposed with those reported for 1A52.

Moreover, we only observed one amino acid change, His524 (1A52) to Phe458 (RmERR), which could induce rearrangements in 3D conformations that may be involved in the interaction with estrogen derivatives molecules. This interaction could also be mediated by the position of H12 in RmERR, which varied compared to the position in 1A52. It has been reported that H12 is an essential structural element of the ligand-binding domain since it behaves as a molecular switch between the active and inactive conformation of the ER [67,68]. These results suggested the possibility of a potential interaction of RmERR with estrogen. Then, to identify if the RmERR can bind specific ligands, we decided to perform an in silico molecular docking analysis. Interestingly, the docking with 17β-estradiol, estrone, estriol, and DES revealed that all estrogenic derivatives consistently found the same binding site in RmERR, stabilizing it by molecular interactions (ionic interactions, hydrogen bonds, hydrophobic contacts, weak hydrogen, and p-p interactions). The negative values of ΔG support these potential interactions predicted for each docking, which suggests a feasible event. 

On the other hand, the main idea behind the characterization of a hormonal receptor that participates in different biological processes of ticks is the search for molecular targets to control tick populations. Considering this fact, we assessed some molecules involved in the negative regulation of RmERR. Therefore, we performed docking analysis with estrogenic antagonists and endocrine disruptors. As expected, the results showed an interaction of tamoxifen and hydroxytamoxifen with RmERR in 9 out of 12 and 9 out of 17 amino acids, respectively. This result suggested that these molecules may prevent the binding of RmERR with its natural ligands. The endocrine disruptor, BPA, only showed a concordance of 2 out of 16 aa shared with the binding site amino acids, suggesting a different interaction with the receptor. Finally, the docking of the estrogenic antagonist and the endocrine disruptor showed ΔG negative values similar to those obtained for the estrogens. Only a few ERRs have been identified in some invertebrates, but none of a tick has been characterized up to now. Thus, we decided to elucidate the phylogenetic relationships of RmERR. As the ERRs from several vertebrate host (including *Bos taurus*) and invertebrates belong to different taxonomic classes, it was expected that they were phylogenetically separated into two clusters, as observed in the reconstructed tree. Particularly in invertebrates, ERRs were grouped in clusters that correspond to the Classes Insecta, Crustacea, and Arachnida, which would suggest that they could have some roles in the gonadal or larval development as it has been reported [69]. However, the ERRs functions in non-mammalian species are poorly understood [58]. The most critical vectors compromising veterinary and human health are in the classes Insecta and Arachnida; although their ERRs could be closely related phylogenetically; interestingly, they are grouped in clusters separated by the Class Crustacea. Whereas the ERRs of different tick species, such as *D. silvarum*, and *Rhipicephalus* spp., are grouped in a clade, those of *I. scapularis* are in a separate clade. Even in *Trichoplax*, the most simplest known animal, an ERR has been reported, which supports the theory that evolutionarily, ERRs could be more ancient than ERs [70].

In this work, the interaction of RmERR with estrogenic compounds is proposed, suggesting that the host blood components, including hormones, could interact with tick estrogenic receptors similar to those reported but probably with different affinity or to another binding site by the process of transregulation. Although this process is well described in several parasitic infections, the occurrence of this intrinsic communication between the host and the tick has not been studied. It is essential to highlight that a possible interaction of vertebrate host steroid hormones with any tick species had never been reported before. In this regard, elucidating these mechanisms opens the possibility of searching for new targets to control ticks and tick-borne diseases.

## Data Availability

The data presented in this study are openly available in NCBI-gene, and KEGG with the reference numbers indicated in the manuscript.

## Supplementary Materials

The following supporting information can be downloaded at: https://www.mdpi.com/article/10.3390/microorganisms11092294/s1, Table S1: Identity percentages of human ER and ERR and RmERR sequences. The identity of RmERR with human ER ranges from 32.78% to 33.33% while the identity with human ERR ranges from 44.87% to 50.28%.
