# Supplementary material for: A Novel Rhipicephalus microplus Estrogen Related Receptor (RmERR), a Molecular and In Silico Characterization of a Potential Protein Binding Estrogen
Source: Microorganisms. 2023 Sep 12;11(9):2294. doi: 10.3390/microorganisms11092294 (PMC10536290; doi:10.3390/microorganisms11092294)
Supplement: Supplementary file 1 [file microorganisms-11-02294-s001.zip › microorganisms-2575850-supplementary.pdf]

Table S1. Identity percentages of human ER and ERR and RmERR sequences. The identity of RmERR with human ER ranges from 32.78% to 33.33% while the identity with human ERR ranges from 44.87% to 50.28%.

	P03372.2 ER alpha _H. sapiens	Q92731.2 ER beta H. sapiens	119164474 ERR R. microplus	KAI4072039.1ERR1 *H. sapiens*	KAI4061772.1ERR2 *H. sapiens*	KAI4084975.1ERR3 *H. sapiens*
P03372.2 ER alpha_*H. sapiens*	100.00	49.05	33.33	35.28	36.26	33.91
Q92731.2 ER beta *H. sapiens*	49.05	100.00	32.78	34.94	33.58	32.56
119164474 ERR *R. microplus*	33.33	32.78	100.00	**50.25**	**44.87**	**45.37**
KAI4072039.1ERR1 *H. sapiens*	35.28	34.94	50.25	100.00	62.22	63.12
KAI4061772.1ERR2 *H. sapiens*	33.91	32.56	45.37	63.12	100.00	78.06
KAI4084975.1ERR3 *H. sapiens*	36.26	33.58	44.87	62.22	78.06	100.00
